# Effect of Unloaded and Curcumin-Loaded Solid Lipid Nanoparticles on Tissue Transglutaminase Isoforms Expression Levels in an Experimental Model of Alzheimer’s Disease

**DOI:** 10.3390/antiox11101863

**Published:** 2022-09-21

**Authors:** Agatina Campisi, Giovanni Sposito, Rosalia Pellitteri, Debora Santonocito, Julia Bisicchia, Giuseppina Raciti, Cristina Russo, Pamela Nardiello, Rosario Pignatello, Fiorella Casamenti, Carmelo Puglia

**Affiliations:** 1 Department of Drug Sciences and Health, University of Catania, 95125 Catania, Italy; 2 CERNUT-Research Centre for Nutraceuticals and Health Products, University of Catania, 95125 Catania, Italy; 3 Institute for Biomedical Research and Innovation (IRIB), National Research Council, 95126 Catania, Italy; 4 NANOMED-Research Center on Nanomedicine and Pharmaceutical Nanotechnology, University of Catania, 95125 Catania, Italy; 5 Department of Biomedical and Biotechnological Sciences, University of Catania, 95125 Catania, Italy; 6 Department of Neuroscience, Psychology, Drug Research and Child Health, University of Florence, 50134 Florence, Italy

**Keywords:** antioxidants, curcumin, tissue transglutaminase, solid lipid nanoparticles, TgCRND8 mice, neuroprotection

## Abstract

Alzheimer’s disease (AD) is a neurodegenerative disease representing the most prevalent cause of dementia. It is also related to the aberrant amyloid-beta (Aβ) protein deposition in the brain. Since oxidative stress is involved in AD, there is a possible role of antioxidants present in the effected person’s diet. Thus, we assessed the effect of the systemic administration of solid lipid nanoparticles (SLNs) to facilitate curcumin (CUR) delivery on TG2 isoform expression levels in Wild Type (WT) and in TgCRND8 (Tg) mice. An experimental model of AD, which expresses two mutated human amyloid precursor protein (APP) genes, was used. Behavioral studies were also performed to evaluate the improvement of cognitive performance and memory function induced by all treatments. The expression levels of Bcl-2, Cyclin-D_1_, and caspase-3 cleavage were evaluated as well. In this research, for the first time, we demonstrated that the systemic administration of SLNs-CUR, both in WT and in Tg mice, allows one to differently modulate TG2 isoforms, which act either on apoptotic pathway activation or on the ability of the protein to repair cellular damage in the brains of Tg mice. In this study, we also suggest that SLNs-CUR could be an innovative tool for the treatment of AD.

## 1. Introduction

Alzheimer’s disease (AD) is a neurodegenerative disease that represents the most prevalent cause of dementia, affecting about 50 million people in the world. It is characterized by cognitive dysfunction, progressive memory loss, language disturbance, and mood–behavioral changes [1]. AD is associated with synaptic failure, oxidative stress, neuroinflammation, and mitochondrial dysfunction [2]. It is also related to the aberrant amyloid-beta (Aβ) protein deposition in the brain, which is a gradual process involving the post-translational modification responsible for fibrillar inclusions and aggregates in the brain [3]. Aβ causes oxidative stress, inflammation, neurotoxicity and the deterioration of the neurotransmission system [4,5]. In addition, it is a substrate of tissue transglutaminase (TG2), a ubiquitarian calcium-dependent protein, which is involved in physiological and pathological processes, including AD [6,7]. TG2 is distributed in many types of tissues, including brain, and is mainly localized in the cytoplasmic compartment of neurons, in nuclei [8], and the extracellular matrix [9]. Depending on its intracellular localization, TG2 plays different functions: in the cytosol, the protein is involved in the apoptotic processes in a stimuli-dependent manner [10]; when it is localized in the nucleus, it phosphorylates some proteins essential for its TG2 kinase activity [11]. In addition, TG2 shows two isoforms, short TG2 (TG2-S) and long TG2 (TG2-L), which have different cellular localizations [12,13,14]. In particular, TG2-S is localized both in the cytosol and in the mitochondria, and its expression levels increase during apoptosis [12,13]. TG2-L is present in the nucleus, playing a protective role during cellular injury and/or apoptosis [12,13].

Our previous findings highlighted that the exposure of olfactory ensheathing cells (OECs)—a particular glial cell type present in the olfactory system—to Aβ(1–42) full native peptide, and its fragments, induced an increase in TG2 expression levels and a different expression pattern of its isoforms [15]. Furthermore, we found that indicaxanthin pretreatment, a phytochemical with antiproliferative and anti-inflammatory activities [16], was able to reduce TG2 overexpression. This would also modulate the TG2 isoform expression and stimulate the reparative role exerted by the protein. We further reported that Aβ induced TG2 conformation change from “open” to “close” status in OECs, thus reversing the effect induced by Aβ. These data show that indicaxanthin pretreatment might stimulate neural regeneration in AD.

Therefore, the involvement of oxidative stress in AD suggests a possible role of antioxidants present in the diet of the effected person [17]. In particular, curcumin (CUR), a natural antioxidant phenolic compound extracted from the rhizome of *Curcuma longa,* and its derivatives, represent promising active compounds for the treatment of AD [18,19,20,21,22].

Several findings demonstrated that CUR shows strong neuro-protective effects improving cognitive function; promoting axonal regeneration [23,24,25,26,27,28]; reducing apoptotic proteins; upregulating the anti-apoptotic protein Bcl-2; and also stimulating neurogenesis and neuroplasticity. In addition, recent data demonstrated that CUR is able to downregulate TG2 overexpression in mouse microglial cells (exposed to lipopolysaccharide or Aβ 1–42 full peptide), thus suggesting its possible use for neuroprotection in neuroinflammatory pathologies, including AD [29]. In vitro and in vivo studies on AD models also demonstrated that CUR was able to inhibit the formation of Aβ oligomers [30,31]. However, in vivo use of CUR is limited because of its low bioavailability in the brain, poor absorption, and rapid systemic clearance [27]. To overcome the unfavorable features of this interesting natural compound, and allow its application for AD treatment, innovative strategies are required [32,33]. An emerging approach to this is the use of nanotechnologies. In particular, solid lipid nanoparticles (SLNs) possess unique physicochemical properties and are able to cross the blood–brain barrier (BBB), representing a promising strategy for the treatment of nervous system disorders, including AD [34]. Notably, in our previous studies, it was demonstrated that encapsulated CUR in SLNs increased its bioavailability and also its residence time in the brain. Furthermore, since the use of SLNs through parenteral administration is limited by the opsonization process (which has a short half-life of approximately 3–5 min), stealth nanoparticles have been realized to avoid the defense line represented by the macrophages. Specifically, stealth SLNs are colloidal carriers made from solid lipids with a modified surface in order to prolong their systemic residence time, and achieve a therapeutic drug concentration at the level of the central nervous system (CNS). This stealth strategy was obtained using a flexible hydrophilic polymer, such as polyethylene glycol (PEG) [35]. Many studies have proved that PEGylation not only stabilizes the SLNs, but also modulates their kinetics of cellular absorption [36,37].

Herein, we assessed the effect of the systemic administration of unloaded and CUR-loaded SLNs on the expression levels of TG2-L and TG2-S in transgenic, hemizygous, CRND8 (Tg) mice (which harbor a double-mutant gene of APP695 with a (C57)/(C57/C3H) genetic background), and in non-Tg hybrid (C57)/(C57/C3H) wild type (WT), control, littermate mice [38]. Empty and SLNs-CUR were formulated and characterized according to the method of Puglia et al. [34]. Behavioral studies were also performed to evaluate the improvement of cognitive performance and memory functions, which were induced by all of the treatments. Furthermore, in order to verify if the treatment with unloaded and CUR-loaded SLNs were able to counteract neuronal death, and the role played by TG2 isoforms, the expression levels of Bcl-2 (a mitochondrial protein with anti-apoptotic activity) was detected. Cyclin-D_1_, a protein involved in the cell progression cycle, and caspase-3 cleavage (the executioner caspase in apoptosis), were also tested.

## 2. Materials and Methods

### 2.1. SLNs Preparation

Curcumin-loaded SLNs (CUR-SLNs) were formulated using the *solvent evaporation* method reported by Santonocito et al. [39] using Compritol^®^ 888 ATO (glycerol behenate; Gattefossè, Milan, Italy) as lipid component and Lutrol F68^®^ (Poloxamer 188; BASF ChemTrade GmbH, Burgbernheim, Germany) as surfactant. Briefly, CUR (99 mg; Merck, St. Louis, MO, USA); injectable soy lecithin (60 mg; Nikko Chemical, Milan, Italy); 1,2-distearoyl-sn-glycero-3-phosphoethanolamine-*N*-[amino(polyethylene glycol)-2000] ammonium salt (DSPE-PEG_2000_, 15 mg; Lipoid GmbH, Ludwigshafen, Germany); and Compritol 888 ATO (60 mg) were solubilized in ethanol (10 mL) at 70 °C. The obtained phase was dispersed in the hot (70 °C) surfactant solution (Lutrol F68, 0.5% *w*/*v*) and cooled in an ice bath (2–3 °C) for 5 min. Finally, ethanol was removed under vacuum. Please note, empty nanoparticles were prepared by the same procedure without adding CUR.

### 2.2. SLNs Characterization

Dynamic light scattering (DLS) and electrophoretic light scattering (ELS) were used for measuring average size (Z-Ave), polydispersity index (PDI), and zeta potential (ZP, ξ), respectively. Analyses were performed at 20 °C using a Zeta Sizer Nano-ZS90 (Malvern Instrument Ltd., Worcs, UK), while using a solid-state laser with a nominal power of 4.5 mW and a maximum output of 5 mW. Each measurement was performed, at least, in triplicate.

### 2.3. Animals and Treatment

**Figure 1:**
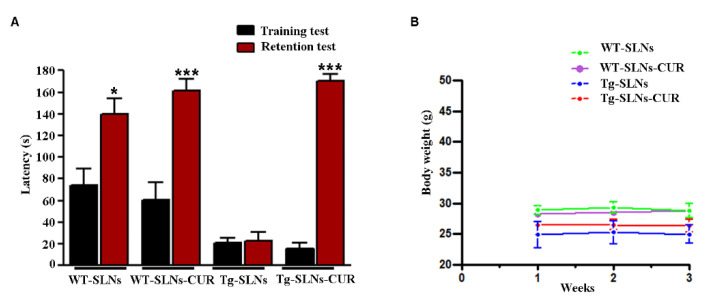
(**A**) Step-down inhibitory avoidance test, the training test (black bars) showed no significant differences between groups. The 24 h retention test (red bars) showed increased latencies in control (WT-SLNs, * *p* < 0.05) and SLNs-CUR-treated WT and Tg mice, when compared to their respective training latencies and to the retention latencies of SLNs Tg mice (*** *p* < 0.001). In untreated Tg mice, retention latencies were not significantly different from training latencies. (**B**) Animal weight during 3 weeks experimental time: SLNs and SLNs-CUR did not affect body weight of WT and Tg mice.

WT and Tg mice (20–30 g) were maintained at 12 h light/dark room at 21–23 °C with tap water and standard chow provided ad libitum. Behavioral experiments and treatments were performed in accordance with the European (2010/63/EC) and National (DL 26/2014, Permit number 931/2020-PR) guidelines in order to minimize the suffering of animals. Four-month-old WT and Tg mice (*n* = 5/group/genotype) were used for the treatment. The animals were divided into four groups and intraperitoneally (i.p.) injected for 3 consecutive weeks, three times a week: (i) WT mice treated with SLNs (*n* = 5 WT, 0.1 mL/10 g); (ii) WT treated with SLNs-CUR (*n* = 5, WT 150 mg/kg); (iii) Tg mice treated with SLNs (*n* = 5 Tg, 0.1 mL/10 g); (iv) Tg treated with SLNs-CUR (*n* = 5 Tg, 150 mg/kg). Additionally, the CTR groups for both WT and Tg mice, treated with saline solution, were used; these results were not reported in Figure 1 because they did not differ from the SLNs CTR groups. The animals were weighed once a day, the body weight recorded, and the dose to be administered i.p. was recalculated according to the body weight.

### 2.4. Behavioral Experiment: Step down Inhibitory Passive Avoidance Task

At the end of the treatment, the memory performance was investigated via a frequently used behavioral “Step down test”. This is based on the animal learning regarding the exploration of a compartment with a foot shock delivered through the floor grid [40]. After exposure to the same environment, the mice will avoid going down, or increase the latency, before going down onto the grid. The word “passive avoidance” indicates in vivo experiments in which the animal learns to avoid a specific event, typically an innate behavior, which is dangerous due to a linked punishment [41]. An animal, when it is located in an open field, spontaneously employs most of its time in dark places, particularly near walls and corners. When it is placed on an elevated platform in the center of a field, the animal will try to leave the platform, explore the environment, and move toward walls. Based on this natural behavior, 60 different variants of the step-down inhibitory avoidance task have been used [42,43]. The apparatus consists of a 50 × 25 × 25 cm Plexiglas box with a steel grid floor through which electric shocks (20 mA, 50 Hz) were delivered. A Plexiglas platform (5 × 8 × 25 cm) was also fixed in the center of the grid floor. For the training test (TT; day 1), each mouse was placed on the platform. When a mouse stepped down with all four paws on the grid floor, intermittent electric shocks were delivered continuously for 3 s. Shock parameters, such as animal vocalization, were recorded in order to select the mice to be used in the next test, i.e., the retention test (RT). Twenty-four hours after training, each mouse was placed on the platform again and the step-down latencies were compared between the TT and RT. An upper cut-off time of 180 s was set. If RT latency was longer than that of the TT it meant that the mouse learnt and remembered the punishment received the day before; on the contrary, however, the mouse showed a memory deficit. At the end of the assay, all of the mice were sacrificed by cervical dislocation and the brains were quickly removed and stored at −80 °C for in vitro experiments.

### 2.5. Isolation of Total Protein and Western Blot Analysis

The brains of all animals were lysed with cell lysis buffer composed of 50 mM Tris-HCl (pH 6.8); 150 mM NaCl; 1 mM EDTA; 0.1 mM PMSF; 10 µg/mL of aprotinin; leupeptin; pepstatin; centrifuged at 13,000× *g* for 10 min at 4 °C; and the supernatants containing total cell proteins were collected [8,44,45]. Subsequently, protein quantification was performed by the bicinchoninic acid method, which was purchased from Thermo-Fisher Company (Monza, Italy). A total of 40 µg of proteins were separated through 4–15% precast SDS–polyacrylamide gels. Filters obtained were then incubated with mouse monoclonal antibody against Bcl-2 (1:500), provided by Abcam (Milan, Italy); rabbit monoclonal antibody against Cyclin D_1_ (1:10,000), supplied by Becton, Dickinson (Florence, Italy); mouse monoclonal antibody against TG2 (1:1000), provided by Abcam (Milan, Italy); mouse monoclonal antibody against Caspase-3 (1:500) obtained by Becton, Dickinson (Florence, Italy); and mouse monoclonal antibody against α-tubulin (1:500), provided by the Thermo-Fisher Company (Monza, Italy). Anti-rabbit IgG horseradish peroxidase-conjugated and anti-mouse IgG horseradish peroxidase-conjugated were then used. The expression of each protein was visualized through Western Lightning Plus-ECL, which was provided by the Thermo-Fisher Company (Monza, Italy), and enhanced via chemiluminescence substrate after autoradiography filter exposure. Blots were then scanned and quantified through a ChemiDoc Imaging System (ChemiDoc™ Imaging System, Bio-Rad, Milan, Italy).

### 2.6. Statistical Analysis

One-way ANOVA with Bonferroni’s post hoc test was used to analyze step down inhibitory avoidance data, and Holm–Sidak’s post hoc test was used to analyze in vitro experiments. Data are reported as mean ± SD of four separated experiments in duplicate, and differences between groups were considered to be significant at * *p* < 0.05.

## 3. Results

### 3.1. SLNs Characterization

SLNs-CUR were formulated following a validated method and all components used for the formulation were found to be safe [39]. DLS data showed technological parameters suitable for parenteral administration: polydispersity index value (PDI) was 0.26; mean diameter (Z-Ave) ranging from 150 to 180 nm; and zeta potential (ZP) values were in the range of −29 to −24 mV (Table 1). As previously reported [39], this latter value predicts a good long-term stability.

### 3.2. Behavioral Experiments

WT and Tg mice were systemically i.p. administered for 3 weeks, three times a week, with unloaded SLNs or SLNs-CUR (150 mg/kg).

We found that both empty SLNs and SLNs-CUR were well tolerated, no evident side effects were revealed and no animal died. To evaluate potential effects of SLNs-CUR on cognitive functions at the end of treatment, the animals were tested for behavioral performance in the step-down test and the data are reported in Figure 1A. To exclude potential SLNs and SLNs-CUR toxicity, the body weight of the animals were recorded during the period of treatment shown in Figure 1B. In the step-down inhibitory avoidance test, no significant differences were observed among all groups during the training test. In the 24 h RT, the step-down latencies recorded with SLNs-Tg mice were significantly reduced in respect to WT groups (*** *p* < 0.001). SLNs-CUR treatment to Tg mice significantly improved their performance (*** *p* < 0.001, vs. SLNs-Tg mice) to levels comparable to those displayed by WT-SLNs and WT-SLN-CUR mice.

This set of experiments highlighted that the systemic administration in Tg mice of SLNs-CUR induced a significant improvement of cognitive performance and memory function, showing that CUR loaded into SLNs was able to explicate a protective and/or reparative function in the brain in an AD mouse model.

### 3.3. Effect of SLNs and SLNs-CUR on TG2 Isoform Expressions

**Figure 2:**
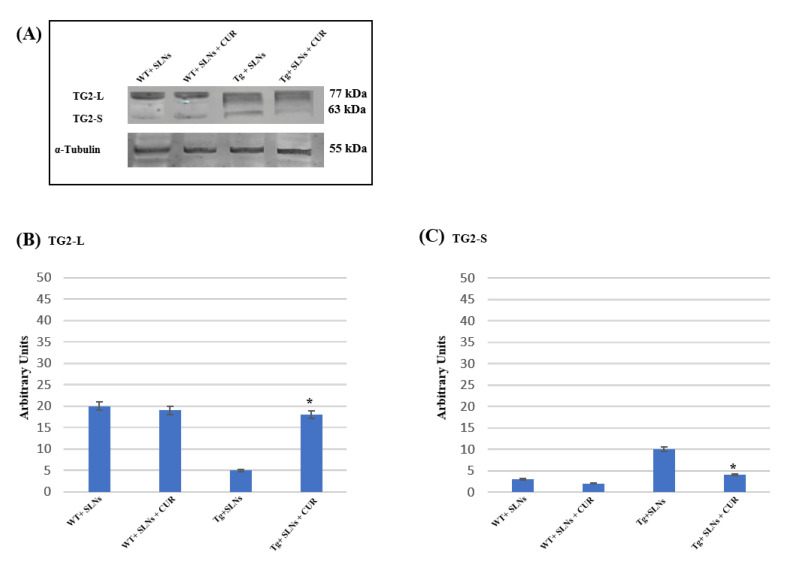
(**A**) Western blotting analysis for both TG2 isomers expression levels in total cellular lysates from WT-SLNs, WT-SLNs-CUR, Tg-SLNs, and Tg-SLNs-CUR mice systemically administrated for 3 weeks. (**B**) Densitometric analysis of TG2-L and (**C**) TG2-S expression relieved after α-tubulin normalization. The results are expressed as the mean ± S.D. of the values of n.5 separate experiments in triplicate. * *p* < 0.05 Tg-SLNs-CUR vs. Tg-SLNs.

Figure 2 shows a representative immunoblot and densitometric analysis related to TG2 isoform expression levels, these were performed in the total cell lysates of WT-SLNs, WT-SLNs-CUR, Tg-SLNs mice, and Tg-SLNs-CUR-treated mice after 3 weeks of systemic administration [12,13,15]. No significant changes in the TG2 isoform expressions, both in WT-SLNs and Tg-SLNs-CUR mice, were observed. In Tg-SLNs mice, TG2-L expression levels (molecular weight 77–78 kDa) appeared at lower levels compared to those observed in WT-SLNs ones. The systemic administration of Tg-SLNs-CUR mice induced a significant increase in TG2-L levels. In contrast, TG2-S (molecular weight 62–63 kDa) was found at low levels in WT-SLNs mice, whereas it was expressed at high levels in Tg-SLNs. Systemic administration of Tg mice with SLNs-CUR enabled an increase in TG2-L expression which appeared at similar values to those found in WT-SLNs-CUR. In parallel, it reduced TG2-S expression at the same levels to those observed in the WT-SLNs-CUR-treated mice. Densitometric analysis, performed after normalization with α-tubulin, confirmed all the results.

These data highlighted that systemic administration of SLNs-CUR, both in the WT and in the Tg mice, differently modulates TG2 isoforms acting on apoptotic pathway activation or on the ability of the protein to repair cellular damage in the brain of Tg mice.

### 3.4. Cyclin-D_1_ Expression Levels

**Figure 3:**
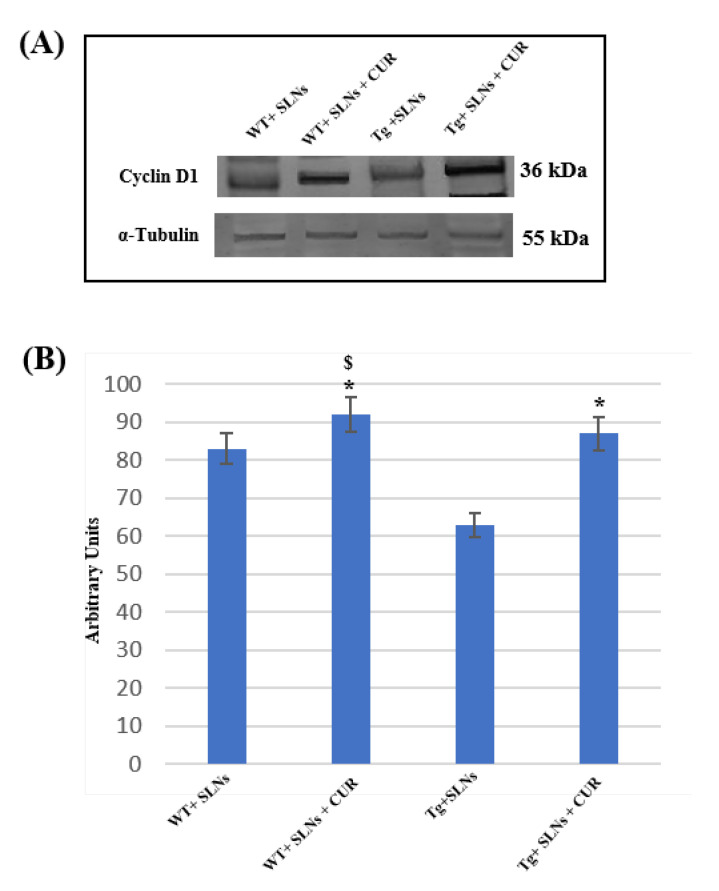
(**A**) Representative immunoblots through Western blotting analysis for Cyclin-D_1_ expression levels in total cellular lysate from WT-SLNs, WT-SLNs-CUR, Tg-SLNs, and Tg-SLNs-CUR mice systemically administrated for 3 weeks. (**B**) Densitometric analysis of Cyclin-D_1_ expression levels performed after normalization with α-tubulin. The results are expressed as the mean ± S.D. of the values of five separate experiments performed in triplicate. * *p* < 0.05, Tg-SLNs-CUR vs. Tg-SLNs; ^$^ *p* < 0.05.

To assess the role played by TG2 in the cellular brain repair, induced by 3 weeks of systemic administration to WT-SLNs, WT-SLNs-CUR, Tg-SLNs mice, and Tg-SLNs-CUR-treated mice, Cyclin-D_1_ protein levels were analyzed through Western Blot (Figure 3).

A slight, but significant, difference between WT-SLNs and WT-SLNs-CUR mice in Cyclin-D_1_ expression levels was observed. This result highlighted that the systemic administration of the animals with SLNs did not induce any toxic effect and that the presence of CUR, stimulating cellular proliferation, might ameliorate brain functions in control mice. In Tg-SLNs mice, Cyclin-D_1_ expression appeared at low levels. SLNs-CUR systemic administration to Tg mice induced a strong increase in Cyclin-D_1_ expression levels, when compared with untreated ones, that were also at similar levels to those found in SLN-WT mice. Data were confirmed by densitometric analysis performed after normalization with α-tubulin.

This set of experiments demonstrated that the systemic administration of CUR-loaded SLNs was able to stimulate TG2 repair activity, activating the stem self-renewal in the brain through the increase in Cyclin-D_1_ expression levels.

### 3.5. Bcl-2 Expression Levels

To verify if SLNs-CUR systemic administration in Tg mice was able to restore the aberrant death stimulus, the impairment of the mitochondria functions and the correlation with TG2 over expression [46], Bcl-2 expression levels were assessed.

**Figure 4:**
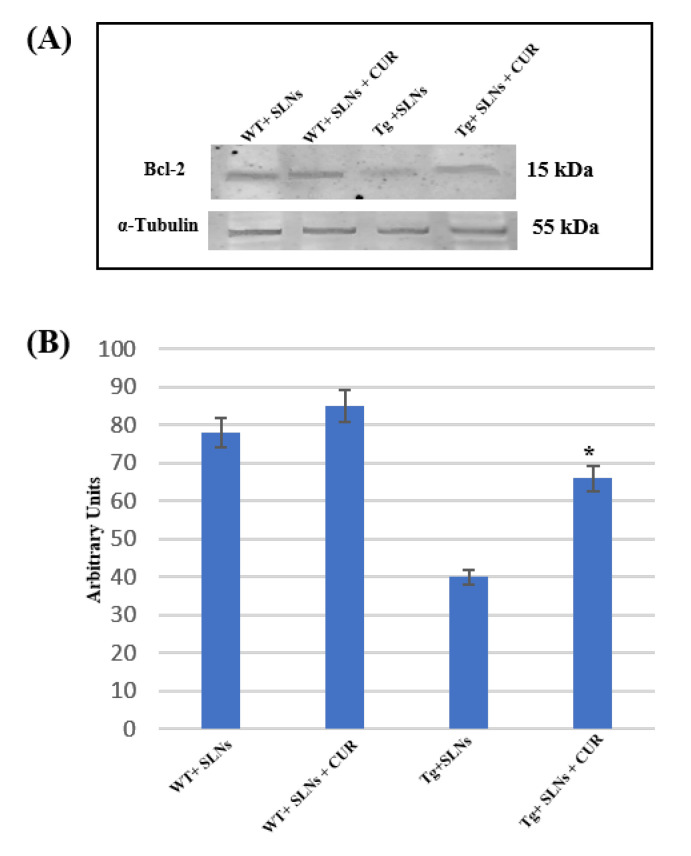
(**A**) Representative immunoblots analysis by Western blotting for Bcl-2 expression levels in total cellular lysate from WT-SLNs, WT-SLNs-CUR, Tg-SLNs, and Tg-SLNs-CUR mice systemically administrated for 3 weeks. (**B**). Bcl-2 expression densitometric analysis obtained after normalization with α-tubulin. The results are expressed as the mean ± S.D. of the values of five separate experiments performed in triplicate. * *p* < 0.05 Tg-SLNs-CUR vs. Tg-SLNs.

Figure 4 shows Western blot and densitometric analysis for Bcl-2, performed on total cellular lysates from WT-SLNs, WT-SLNs-CUR, Tg-SLNs, and SLNs-CUR-Tg-treated mice. Notably, Bcl-2 expression levels appeared at low levels in Tg mice.

No significant changes between WT-SLNs and WT-SLNs-CUR mice in Bcl-2 expression levels were found. This data showed that the systemic administration of SLNs did not induce mitochondrial damage. In Tg-SLNs mice, Bcl-2 expression appeared at low levels. The administration of SLNs-CUR in Tg mice induced a strong increase in Bcl-2 expression levels that, when compared with untreated ones, were also similar to those found in WT-SLNs mice. Data were confirmed by densitometric analysis, which was performed after normalization with α-tubulin.

This set of experiments highlighted that treatment with SLNs-CUR was able to restore the mitochondrial functions modified in Tg mice.

### 3.6. Caspase-3 Cleavage

**Figure 5:**
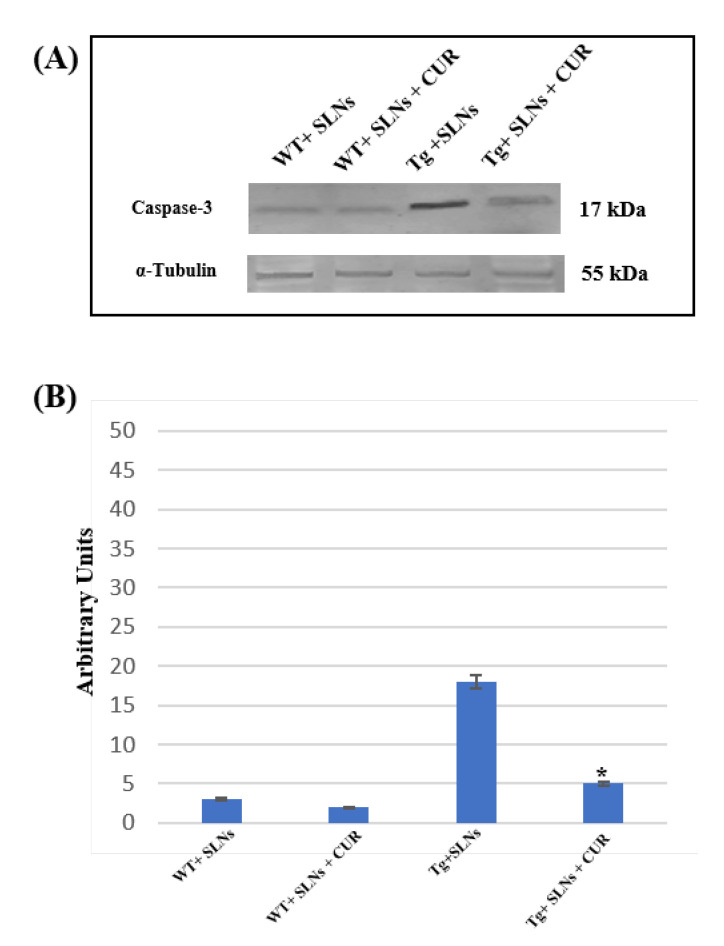
(**A**) Caspase-3 cleavage in total cellular lysate from WT-SLNs, WT-SLNs-CUR, Tg-SLNs, and Tg-SLNs-CUR mice systemically administrated for 3 weeks relieved by Western blot. (**B**) Densitometric analysis of caspase-3 cleavage expression performed after α-tubulin normalization. The results are expressed as the mean ± S.D. of the values of five separate experiments performed in triplicate. * *p* < 0.05 Tg-SLNs-CUR vs. Tg-SLNs.

To verify the TG2-mediated apoptotic pathway in Tg-SLNs and in Tg-SLNs-CUR mice, caspase-3 cleavage through Western Blot analysis was performed and evaluated to total cell lysates from WT-SLNs, Tg-SLNs, and Tg-SLNs-CUR mice (Figure 5). No significant change in caspase-3 cleavage, both in WT-SLNs-treated and WT-SLNs-CUR mice, was observed. This result highlighted that the systemic administration of SLNs did not activate the apoptotic pathway, thus confirming that they are not toxic. High levels of caspase-3 cleavage in Tg-SLNs mice were found, however. Moreover, systemic administration of Tg-SLNs-CUR induced a strong decrease in caspase-3 cleavage when compared with those observed in the Tg-SLNs mice, even if its levels appeared slightly higher than those found in the WT-SLNs-CUR-treated mice.

These findings highlighted the role played by TG2 in the control of apoptotic pathway activation in the brain of mice treated with unloaded-SLNs and loaded-SLNs-CUR.

## 4. Discussion

The aim of this study was to assess the effect of the systemic administration of unloaded and CUR-loaded SLNs on TG2-L and TG2-S expression in WT and Tg mice. In particular, Tg mice used for these studies expressed two mutated human APP genes, which are implicated in AD, thus showing a very rapid development of amyloid pathology due to the deposition of Aβ, which are responsible for amyloid plaques in the cerebral cortex and hippocampus, within three months of age [38]. To verify the improvement of cognitive performance and memory function induced by the treatments, behavioral studies were performed. In addition, the expression levels of Bcl-2, a mitochondrial protein with anti-apoptotic activity, were evaluated in order to verify if the treatment with SLNs-CUR was able to counteract neuronal death and the role played by TG2 isoforms. Cyclin-D_1_, a protein involved in the cell progression cycle, and caspase-3 cleavage, the executioner caspase in apoptosis, were also tested.

In our previous studies, we demonstrated that TG2 is overexpressed in OECs exposed to Aβ(1–42) and its toxic fragment Aβ(25–35), and that the treatment of the cells with indicaxanthin—an antioxidant from the *Opuntia ficus-indica* fruit—was able to counteract Aβ effects [15]. TG2, a calcium-dependent protein with transamidating activity involved in AD, contributes to the formation of amyloid aggregates responsible for the alterations of several proteins [6] and a dysregulation of the autophagy process [47].

In this research, for the first time, we demonstrate that the systemic administration of SLNs-CUR, both in WT and in Tg mice, can differently modulate TG2 isoforms acting on apoptotic pathway activation or on the ability of the protein to repair cellular damage in the brains of Tg mice. In particular, systemic administration of Tg-SLNs-CUR mice induced a significant increase in TG2-L levels. TG2 isoforms play a role in the control of cell proliferation, the regulation gene expression, cell survival, and cellular differentiation [14,44,48]. In contrast, TG2-S, exerting transamidating activity and acting as an apoptotic factor [10,44], was found at low levels in WT-SLNs mice, whereas it was expressed at high levels in Tg-SLNs. To verify the protective role played by TG2-L in Tg mice (when administrated with SLNs-CUR Cyclin-D_1_), a marker of cellular proliferation was evaluated [49]. In parallel, a significant reduction in TG2-S expression levels in Tg-SLNs-CUR-treated mice was observed. A significant increase in Bcl-2 expression accompanied by a decrease in caspase-3 cleavage was also found. These last results demonstrate that the treatment of Tg mice with SLNs-CUR for three weeks can reduce the formation of an alternative splice variant of TG2, which is responsible for apoptotic activation and cell death. Furthermore, in vivo data support our previous observations, performed on OEC cultures exposed to Aβ, both in the absence and in the presence of indicaxanthin [15]. These results also suggest that SLNs-CUR treatment in an AD experimental model could achieve TG2 conformation change from “open state” during the damage to “close state” which is involved in the stimulating of neural repair.

Behavioral results confirmed that the systemic administration of SNLs-CUR in Tg mice induced a significant improvement of cognitive performance and memory function, contributing also to the repair of cellular damage in transgenic mice.

## 5. Conclusions

Our results provide clear evidence that the systemic administration of curcumin-loaded SLNs in TgCRND8 mice can reduce TG2-S expression, therefore decreasing apoptotic pathway activation. At the same time, it was able to increase TG2-L levels, which play the reparative role in an AD experimental model. Therefore, further studies are underway to better understand the effect of SLNs-CUR on AD. Moreover, the administration of this nanoformulation could represent a promising and innovative tool for the treatment of this pathology.

## Figures and Tables

**Table 1:** The values of Z-Ave, PDI, and Z-potential for unloaded and CUR-SLN recorded at 20 °C.

Formulation	Z-Ave (nm ± SD)	PDI (−) ± SD	ZP (mV ± SD)
Blank SLNs	176.0 ± 0.2	0.26 ± 0.1	−29.0 ± 0.1
SLNs-CUR	153.3 ± 0.2	0.26 ± 0.2	−23.9 ± 0.1

## Data Availability

The data presented in this study are available in the article.

## References

[1] Mehla J., Gupta P., Pahuja M., Diwan D., Diksha D. (2020). Indian Medicinal Herbs and Formulations for Alzheimer’s Disease, from Traditional Knowledge to Scientific Assessment. Brain. Sci..

[2] Querfurth H.W., LaFerla F.M. (2010). Alzheimer’s disease. N. Engl. J. Med..

[3] Nisbet R.M., Götz J. (2018). Amyloid-β and tau in Alzheimer’s disease: Novel pathomechanisms and non-pharmacological treatment strategies. J. Alzheimer’s Dis..

[4] Sahlin C., Pettersson F.E., Nilsson L.N., Lannfelt L., Johansson A.S. (2007). Docosahexaenoic acid stimulates non-amyloidogenic APP processing resulting n reduced Abeta levels in cellular models of Alzheimer’s disease. Eur. J. Neurosci..

[5] Sun X., Chen W.D., Wang Y.D. (2015). β-Amyloid: The key peptide in the pathogenesis of Alzheimer’s disease. Front. Pharmacol..

[6] Lesort M., Tucholski J., Miller M.L., Johnson G.V. (2000). Tissue transglutaminase: A possible role in neurodegenerative diseases. Prog. Neurobiol..

[7] Wilhelmus M., Jongenelen C.A., Bol J., Drukarch B. (2020). Interaction between tissue transglutaminase and amyloid-beta: Protein-protein binding versus enzymatic crosslinking. Anal. Biochem..

[8] Campisi A., Caccamo D., Li Volti G., Currò M., Parisi G., Avola R., Vanella A., Ientile R. (2004). Glutamate-evoked redox state alterations are involved in tissue transglutaminase upregulation in primary astrocyte cultures. FEBS Lett..

[9] Kuo T.F., Tatsukawa H., Kojima S. (2011). New insights into the functions and localization of nuclear transglutaminase 2. FEBS J..

[10] Milakovic T., Tucholski J., McCoy E., Johnson G.V. (2004). Intracellular localization and activity state of tissue transglutaminase differentially impacts cell death. J. Biol. Chem..

[11] Mishra S., Melino G., Murphy L.J. (2007). Transglutaminase 2 kinase activity facilitates protein kinase A-induced phosphorylation of retinoblastoma protein. J. Biol. Chem..

[12] Citron B.A., Suo Z., SantaCruz K., Davies P.J., Qin F., Festoff B.W. (2002). Protein crosslinking, tissue transglutaminase, alternative splicing and neurodegeneration. Neurochem. Int..

[13] Antonyak M.A., Jansen J.M., Miller A.M., Ly T.K., Endo M., Cerione R.A. (2006). Two isoforms of tissue transglutaminase mediate opposing cellular fates. Proc. Natl. Acad. Sci. USA.

[14] Singh G., Zhang J., Ma Y., Cerione R.A., Antonyak M.A. (2016). The Different Conformational States of Tissue Transglutaminase Have Opposing Affects on Cell Viability. J. Biol. Chem..

[15] Campisi A., Raciti G., Sposito G., Grasso R., Chiacchio M.A., Spatuzza M., Attanzio A., Chiacchio U., Tesoriere L., Allegra M. (2021). Amyloid-Beta Induces Different Expression Pattern of Tissue Transglutaminase and Its Isoforms on Olfactory Ensheathing Cells: Modulatory Effect of Indicaxanthin. Int. J. Mol. Sci..

[16] Allegra M., Tutone M., Tesoriere L., Almerico A.M., Culletta G., Livrea M.A., Attanzio A. (2019). Indicaxanthin, a multi-target natural compound from Opuntia ficus-indica fruit: From its poly-pharmacological effects to biochemical mechanisms and molecular modelling studies. Eur. J. Med. Chem..

[17] Mehla J., Pahuja M., Dethe S.M., Agarwal A., Gupta Y.K. (2012). Amelioration of intracerebroventricular streptozotocin induced cognitive impairment by Evolvulus alsinoides in rats: In vitro and in vivo evidence. Neurochem. Int..

[18] Khanna S., Park H.A., Sen C.K., Golakoti T., Sengupta K., Venkateswarlu S., Roy S. (2009). Neuroprotective and antiinflammatory properties of a novel demethylated curcuminoid. Antioxid. Redox. Signal..

[19] Chainoglou E., Hadjipavlou-Litina D. (2019). Curcumin analogues and derivatives with anti-proliferative and anti-inflammatory activity: Structural characteristics and molecular targets. Expert. Opin. Drug Discov..

[20] Akaishi T., Abe K. (2018). CNB-001, a synthetic pyrazole derivative of curcumin, suppresses lipopolysaccharide-induced nitric oxide production through the inhibition of NF-κB and p38 MAPK pathways in microglia. Eur. J. Pharmacol..

[21] Fang L., Gou S., Liu X., Cao F., Cheng L. (2014). Design, synthesis and anti-Alzheimer properties of dimethylaminomethyl-substituted curcumin derivatives. Bioorg. Med. Chem. Lett..

[22] Venigalla M., Sonego S., Gyengesi E., Sharman M.J., Münch G. (2018). Novel promising therapeutics against chronic neuroinflammation and neurodegeneration in Alzheimer’s disease. Neurochem. Ireddynt..

[23] Kumar A., Naidu P.S., Seghal N., Padi S.S. (2007). Neuroprotective effects of resveratrol against intracerebroventricular colchicine-induced cognitive impairment and oxidative stress in rats. Pharmacology.

[24] Kumar A., Dogra S., Prakash A. (2009). Protective effect of curcumin (*Curcuma longa*), against aluminium toxicity: Possible behavioral and biochemical alterations in rats. Behav. Brain. Res..

[25] Reeta K.H., Mehla J., Gupta Y.K. (2010). Curcumin ameliorates cognitive dysfunction and oxidative damage in phenobarbitone and carbamazepine administered rats. Eur. J. Pharmacol..

[26] Barzegar A., Moosavi-Movahedi A.A. (2011). Intracellular ROS Protection Efficiency and Free Radical-Scavenging Activity of Curcumin. PLoS ONE.

[27] Tsai Y.M., Chien C.F., Lin L.C., Tsai T.H. (2011). Curcumin and its nano-formulation: The kinetics of tissue distribution and blood-brain barrier penetration. Int. J. Pharm..

[28] Desai P.P., Patravale V.B. (2018). Curcumin Cocrystal Micelles-Multifunctional Nanocomposites for Management of Neurodegenerative Ailments. J. Pharm. Sci..

[29] Gatta N.G., Parente A., Guida F., Maione S., Gentile V. (2021). Neuronutraceuticals Modulate Lipopolysaccharide- or Amyloid-β 1-42 Peptide-Induced Transglutaminase 2 Overexpression as a Marker of Neuroinflammation in Mouse Microglial Cells. Appl. Sci..

[30] Reddy P.H., Manczak M., Yin X., Grady M.C., Mitchell A., Tonk S., Kuruva C.S., Bhatti J.S., Kandimalla R., Vijayan M. (2018). Protective Effects of Indian Spice Curcumin Against Amyloid-β in Alzheimer’s Disease. J. Alzheimer’s Dis..

[31] Bisceglia F., Seghetti F., Serra M., Zusso M., Gervasoni S., Verga L., Vistoli G., Lanni C., Catanzaro M., De Lorenzi E. (2019). Prenylated Curcumin Analogues as Multipotent Tools To Tackle Alzheimer’s Disease. ACS Chem. Neurosci..

[32] Puglia C., Santonocito D. (2019). Cosmeceuticals: Nanotechnology-based strategies for the delivery of phytocompounds. Curr. Pharm. Des..

[33] Bonaccorso A., Pellitteri R., Ruozi B., Puglia C., Santonocito D., Pignatello R., Musumeci T. (2021). Curcumin loaded polymeric vs. lipid nanoparticles: Antioxidant effect on normal and hypoxic olfactory ensheathing cells. Nanomaterials.

[34] Puglia C., Pignatello R., Fuochi V., Furneri P.M., Lauro M.R., Santonocito D., Cortesi R., Esposito E. (2019). Lipid nanoparticles and active natural compounds: A perfect combination for pharmaceutical applications. Curr. Med. Chem..

[35] Santonocito D., Granata G., Geraci C., Panico A., Siciliano E.A., Raciti G., Puglia C. (2020). Carob seeds: Food waste or source of bioactive compounds?. Pharmaceutics.

[36] Gref R., Minamitake Y., Peracchia M.T., Trubetskoy V., Torchilin V., Langer R. (1994). Biodegradable long circulating polymeric nanospheres. Science.

[37] Kommareddy S., Amiji M. (2007). Biodistribution and pharmacokinetic analysis of long-circulating thiolated gelatin nanoparticles following systemic administration in breast cancer-bearing mice. J. Pharm. Sci..

[38] Chishti M.A., Yang D.S., Janus C., Phinney A.L., Horne P., Pearson J., Strome R., Zuker N., Loukides J., French J. (2001). Early-onset amyloid deposition and cognitive deficits in transgenic mice expressing a double mutant form of amyloid precursor protein 695. J. Biol. Chem..

[39] Santonocito D., Sarpietro M.G., Carbone C., Panico A., Campisi A., Siciliano E.A., Sposito G., Castelli F., Puglia C. (2020). Curcumin containing pegylated solid lipid nanoparticles for systemic administration: A preliminary study. Molecules.

[40] Bilia A.R., Nardiello P., Piazzini V., Leri M., Bergonzi M.C., Bucciantini M., Casamenti F. (2019). Successful Brain Delivery of Andrographolide Loaded in Human Albumin Nanoparticles to TgCRND8 Mice, an Alzheimer’s Disease Mouse Model. Front. Pharmacol..

[41] Netto C.A., Izquierdo I. (1985). On how passive is inhibitory avoidance. Behav. Neural. Biol..

[42] Kubanis P., Zornetzer S.F. (1981). Age-related behavioral and neurobiological changes: A review with an emphasis on memory. Behav. Neural. Biol..

[43] Chorover S.L., Schiller P.H. (1965). Short-term retrograde amnesia in rats. J. Comp. Physiol Psychol..

[44] Pellitteri R., Bonfanti R., Spatuzza M., Cambria M.T., Ferrara M., Raciti G., Campisi A. (2017). Effect of Some Growth Factors on Tissue Transglutaminase Overexpression Induced by β-Amyloid in Olfactory Ensheathing Cells. Mol. Neurobiol..

[45] Grasso R., Dell’Albani P., Carbone C., Spatuzza M., Bonfanti R., Sposito G., Puglisi G., Musumeci F., Scordino A., Campisi A. (2020). Synergic pro-apoptotic effects of Ferulic Acid and nanostructured lipid carrier in glioblastoma cells assessed through molecular and Delayed Luminescence studies. Sci. Rep..

[46] Piacentini M., Amendola A., Ciccosanti F., Falasca L., Farrace M.G., Mastroberardino P.G., Nardacci R., Oliverio S., Piredda L., Rodolfo C. (2005). Type 2 transglutaminase and cell death. Prog. Exp. Tumor. Res..

[47] Mputhia Z., Hone E., Tripathi T., Sargeant T., Martins R., Bharadwaj P. (2019). Autophagy Modulation as a Treatment of Amyloid Diseases. Molecules.

[48] Tatsukawa H., Furutani Y., Hitomi K., Kojima S. (2016). Transglutaminase 2 has opposing roles in the regulation of cellular functions as well as cell growth and death. Cell. Death Dis..

[49] Hosseini S., Chamani J., Sinichi M., Bonakdar A.M., Azad Z., Ahangari N., Rahimi H.R. (2019). The effect of nanomicelle curcumin, sorafenib, and combination of the two on the cyclin D1 gene expression of the hepatocellular carcinoma cell line (HUH7). Iran. J. Basic. Med. Sci..

